# Comparison of Eleven RNA Extraction Methods for Poliovirus Direct Molecular Detection in Stool Samples

**DOI:** 10.1128/spectrum.04252-22

**Published:** 2023-03-20

**Authors:** Joyce Odeke Akello, Erika Bujaki, Alexander G. Shaw, Adnan Khurshid, Yasir Arshad, Catherine Troman, Manasi Majumdar, Áine O'Toole, Andrew Rambaut, Muhammad Masroor Alam, Javier Martin, Nicholas C. Grassly

**Affiliations:** a Department of Infectious Disease Epidemiology, Imperial College London, London, United Kingdom; b Division of Vaccines, National Institute for Biological Standards and Control (NIBSC), MHRA, Potters Bar, United Kingdom; c Department of Virology, National Institute for Health, Islamabad, Pakistan; d Institute of Evolutionary Biology, University of Edinburgh, Ashworth Laboratories, Edinburgh, United Kingdom; Hôpital Saint-Louis

**Keywords:** PCR amplification, RNA extraction, molecular detection, poliovirus

## Abstract

Direct detection by PCR of poliovirus RNA in stool samples provides a rapid diagnostic and surveillance tool that can replace virus isolation by cell culture in global polio surveillance. The sensitivity of direct detection methods is likely to depend on the choice of RNA extraction method and sample volume. We report a comparative analysis of 11 nucleic acid extraction methods (7 manual and 4 semiautomated) for poliovirus molecular detection using stool samples (*n* = 59) that had been previously identified as poliovirus positive by cell culture. To assess the effect of RNA recovery methods, extracted RNA using each of the 11 methods was tested with a poliovirus-specific reverse transcription-quantitative PCR (RT-qPCR), a pan-poliovirus RT-PCR (near-whole-genome amplification), a pan-enterovirus RT-PCR (entire capsid region), and a nested VP1 PCR that is the basis of a direct detection method based on nanopore sequencing. We also assessed extracted RNA integrity and quantity. The overall effect of extraction method on poliovirus PCR amplification assays tested in this study was found to be statistically significant (*P* < 0.001), thus indicating that the choice of RNA extraction method is an important component that needs to be carefully considered for any diagnostic based on nucleic acid amplification. Performance of the methods was generally consistent across the different assays used. Of the 11 extraction methods tested, the MagMAX viral RNA isolation kit used manually or automatically was found to be the preferable method for poliovirus molecular direct detection considering performance, cost, and processing time.

**IMPORTANCE** Poliovirus, the causative agent of poliomyelitis, is a target of global eradication led by the World Health Organization since 1988. Direct molecular detection and genomic sequencing without virus propagation in cell culture is arguably a critical tool in the final stages of polio eradication. Efficient recovery of good-quality viral RNA from stool samples is a prerequisite for direct detection by nucleic acid amplification. We tested 11 nucleic acid extraction methods to identify those facilitating sensitive, fast, simple, and cost-effective extraction, with flexibility for manual and automated protocols considered. Several different PCR assays were used to compare the recovered viral RNA to test suitability for poliovirus direct molecular detection. Our findings highlight the importance of choosing a suitable RNA extraction protocol and provide useful information to diagnostic laboratories and researchers facing the choice of RNA extraction method for direct molecular virus detection from stool.

## INTRODUCTION

Poliovirus (PV) surveillance is vital to the Global Polio Eradication Initiative (GPEI) effort to eradicate and contain all wild, vaccine-related, and Sabin polioviruses. Surveillance operates through both the detection of the virus in the stools of children suffering from acute flaccid paralysis (AFP) (1) and from wastewater (environmental surveillance) (2). Testing of samples is performed within the Global Polio Laboratory Network (GPLN), and the sensitivity and robustness of methods for poliovirus detection are critical, especially in the late stages of polio eradication. The current detection algorithm is highly sensitive and relies on culture of the virus within susceptible cell lines (3). However, direct detection methods that improve the efficiency of poliovirus detection in terms of speed and eliminate the need for virus isolation by cell culture, in line with containment targets (4), have been developed (5–10). Recently, a method for poliovirus typing/characterization through direct detection by nanopore sequencing (DDNS) has been shown to offer a rapid, accurate, and high-throughput test in a cost-effective manner (6).

Nucleic acid extraction marks a starting point in any molecular method and serves as a key step to recover viral nucleic acid and remove unidentified constituents that inhibit downstream molecular applications (11, 12). Direct detection of viruses from stool samples in particular can be challenging due to the range of inhibitors, such as polysaccharides and bile salts, that interfere with nucleic acid amplification assays. The performance of methods for the removal of these inhibitors can vary (13, 14); hence, the choice of extraction methods is critical for optimal performance of the direct detection molecular assays. Moreover, different nucleic acid extraction methods and kits have been recognized to have various viral RNA recovery efficiencies (14, 15).

Therefore, different RNA extraction methods were compared with a view to identify if specific methods produced better results for different poliovirus molecular detection assays, such as quantitative PCR (qPCR), long-range reverse transcription-PCR (RT-PCR), and suitability for poliovirus direct detection by the DDNS method (6).

The aim of the study was to compare 11 extraction methods (7 manual methods and 4 semi-automated methods) for their ability to efficiently recover poliovirus RNA from stool for subsequent poliovirus direct detection. Performance of the 11 extraction methods was evaluated in two different laboratories in terms of their ability to detect polioviruses in stool suspensions, as determined by poliovirus-specific reverse transcription-quantitative PCR (RT-qPCR), a near-whole-genome amplification pan-poliovirus (PanPV) RT-PCR, pan-enterovirus (PanEV) RT-PCR amplifying the entire capsid, and nested VP1 PCR amplifying the full VP1 region. The QIAamp viral RNA minikit is a commonly used and accepted RNA extraction method in the GPLN and has been used on all the samples included in this study; therefore, it was used as a comparator for all results.

We examined the effect of the extraction methods on these PCR amplification assays and how they differ in their ability to efficiently recover poliovirus genome copies present in stool suspensions. The viral RNA recovered by each of the 11 extraction methods was also assessed for quality based on RNA integrity number equivalent (RINe) and quantity (concentration).

## RESULTS

### Quality and quantity of RNA recovered by 11 extraction methods.

The mean RNA concentration of the 11 extraction methods ranged from 1.54 ± 3.60 ng/μL to 3.30 ± 4.51 ng/μL. The mean RINe (a quality metric used to numerically assess RNA degradation considering the 18S to 28S ribosomal band ratio in relation to the background signal to evaluate the integrity of eukaryotic total RNA [16, 17]) values of the 11 extraction methods ranged from 1.68 ± 0.39 to 3.78 ± 1.93. The highest RNA concentration on average was found in samples extracted with the MagMAX viral RNA isolation kit (manual) and KingFisher MagMAX pathogen RNA/DNA kit (large volume [LV]), while extraction with the KingFisher MagMAX viral RNA isolation kit and Zymo quick viral RNA kit (protocol B) resulted in the lowest average concentration (Table 1). The KingFisher MagMAX pathogen RNA/DNA kits and the KingFisher MagMAX viral RNA isolation kit had, on average, better RINe values, indicating better RNA quality, while the High Pure viral RNA kit without proteinase K (Roche, protocol A) and Zymo quick viral RNA kit (protocol B) had the lowest average RINe values, indicating low RNA quality (Table 1) in comparison to the other methods tested. The Zymo quick viral RNA kit (protocol B) generated the most extractions where RINe and concentration readings could not be determined (Table 1). There was no relation observed between RINe and samples with a nested VP1, PanEV, or PanPV RT-PCR-positive result (*P* = 0.251, *P* = 0.199, and *P* = 0.472, respectively). However, RINe was found to be statistically significant in determining RT-qPCR PV results (*P* < 0.001).

**TABLE 1:** Summary of RNA concentration and integrity for all samples recovered by the 11 extraction methods

Extraction	Total stool samples tested	PV strains contained in samples^*a*^	Percentage of samples with RNA concn	Mean RNA concn ± SD (ng/μL)	Percentage of samples with RINe	Mean RINe ± SD
AllPrep PowerFecal DNA/RNA kit	28	SL1 and SL3	100 (28/28)	2.61 ± 7.06	100 (28/28)	2.27 ± 0.54
High Pure viral RNA kit without proteinase K (Roche protocol A)	28	SL1 and SL3	100 (28/28)	1.65 ± 2.74	100 (28/28)	1.68 ± 0.39
High Pure viral RNA kit with proteinase K (Roche protocol B)	31	PV2, SL1, and SL3	100 (31/31)	2.07 ± 3.71	83.87 (26/31)	2.3 ± 0.60
KingFisher MagMAX viral RNA isolation kit	28	SL1 and SL3	100 (28/28)	1.54 ± 3.60	100 (28/28)	3.55 ± 1.66
KingFisher MagMAX pathogen RNA/DNA kit (SV)	28	SL1 and SL3	100 (28/28)	1.66 ± 2.71	100 (28/28)	3.43 ± 1.86
KingFisher MagMAX pathogen RNA/DNA kit (MV)	28	SL1 and SL3	100 (28/28)	2.22 ± 4.87	100 (28/28)	3.78 ± 1.93
KingFisher MagMAX pathogen RNA/DNA kit (LV)	16	SL1 and SL3	100 (16/16)	3.10 ± 5.47	100 (16/16)	2.56 ± 1.31
MagMAX viral RNA isolation kit (manual)	58	PV2, SL1, and SL3	100 (58/58)	3.30 ± 4.51	89.8 (53/58)	2.90 ± 1.39
QIAamp viral RNA minikit	59	PV2, SL1, and SL3	89.8 (53/59)	2.73 ± 8.20	84.7 (50/59)	2.19 ± 0.84
Zymo quick viral RNA kit (protocol A)	28	SL1 and SL3	100 (28/28)	2.72 ± 9.45	100 (28/28)	2.58 ± 0.96
Zymo quick viral RNA kit (protocol B)	31	PV2, SL1, and SL3	45.2 (14/31)	1.62 ± 2.65	32.3 (10/31)	1.9 ± 0.57

a PV2, Sabin-like type 2 poliovirus; SL1, Sabin-like 1 poliovirus; SL3, Sabin-like 3 poliovirus.

### Poliovirus detection by RT-qPCR after extraction with 11 different methods.

**FIG 1:**
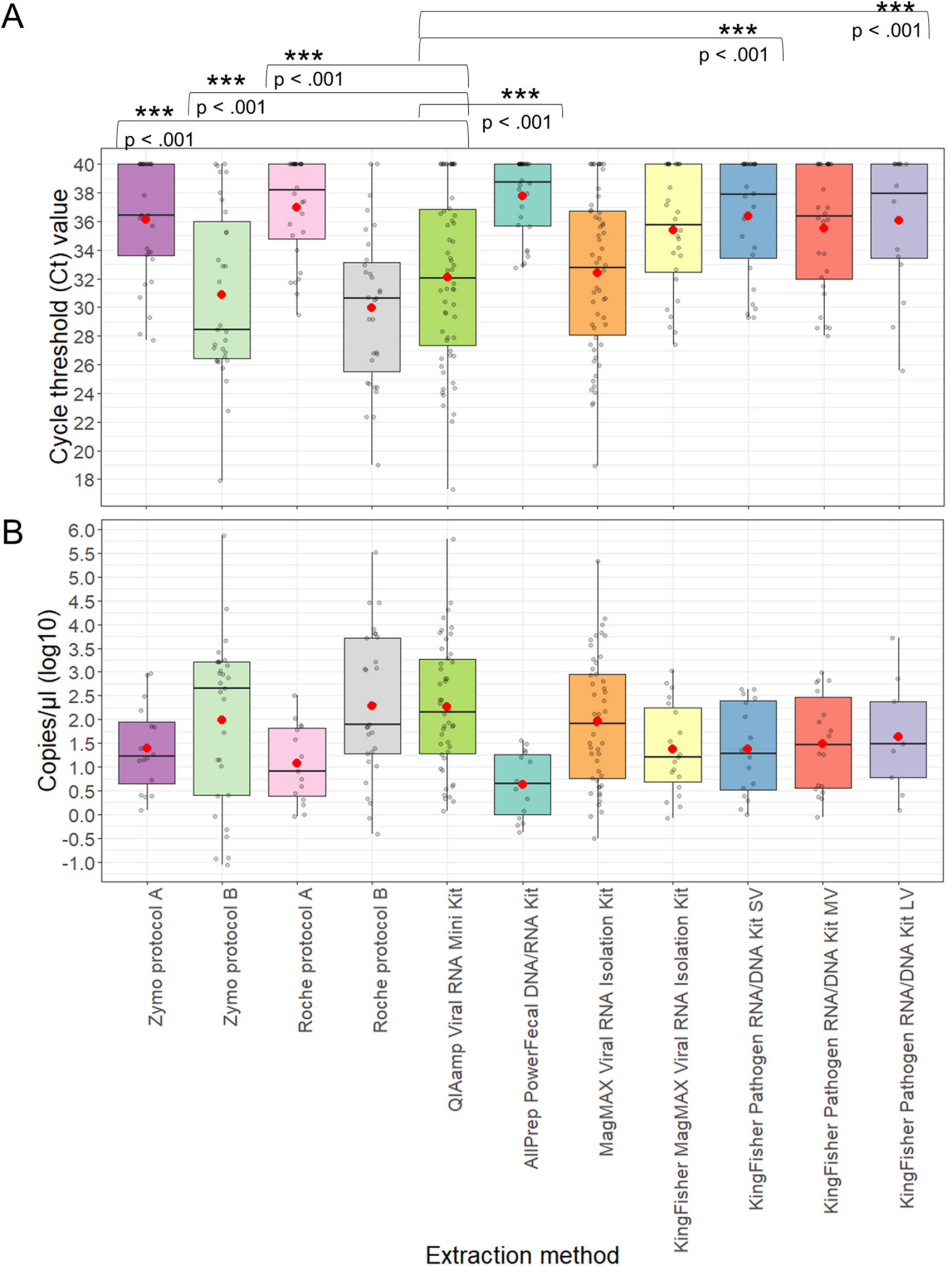
Evaluation of viral RNA recovery for PV positive stool samples by 11 extraction methods. (A and B) Box plots of cycle threshold (*C_T_*) value (A) and viral genome copies/μL (B) of the PV stool samples according to each extraction method. Extracted RNA was tested by RT-qPCR assay using PV specific primers and probe. Each gray circle represents a sample. Each stool sample was tested in the RT-qPCR in duplicates run in parallel with Sabin 1 standards. The box plots show the mean (represented by red dot), median, lower quartile, and upper quartile of the data points for samples extracted by each method, and the whiskers show the range. *P* values are based on a linear mixed effects regression analysis.

Of all extraction methods compared to the QIAamp viral RNA minikit method, samples extracted using the High Pure viral RNA kit with proteinase K (Roche protocol B) had the lowest average cycling threshold (*C_T_*) value (30.0 ± 5.38) and highest mean copies/μL in log_10_ (2.29 ± 1.52). Samples extracted using the AllPrep PowerFecal DNA/RNA kit had the highest average *C_T_* value (37.8 ± 2.69) and lowest mean copies/μL in log_10_ (0.634 ± 0.68) (Fig. 1A and B). The overall effect of extraction method was statistically significant in determining RT-qPCR results compared to the QIAamp viral RNA minikit extraction method (*P* < 0.001). For both *C_T_* values and log viral copies, statistically significant and positive differences were observed for most methods except for the KingFisher MagMAX viral RNA isolation kit, MagMAX viral RNA isolation kit (manual), KingFisher MagMAX pathogen RNA/DNA kit (medium volume [MV]), and High Pure viral RNA kit (Roche, protocol B), which were statistically nonsignificant and positive compared to the QIAamp viral RNA minikit extraction method (Fig. 1).

### Variation in the proportion of PV-positive samples following RNA extraction by 11 different methods.

**FIG 2:**
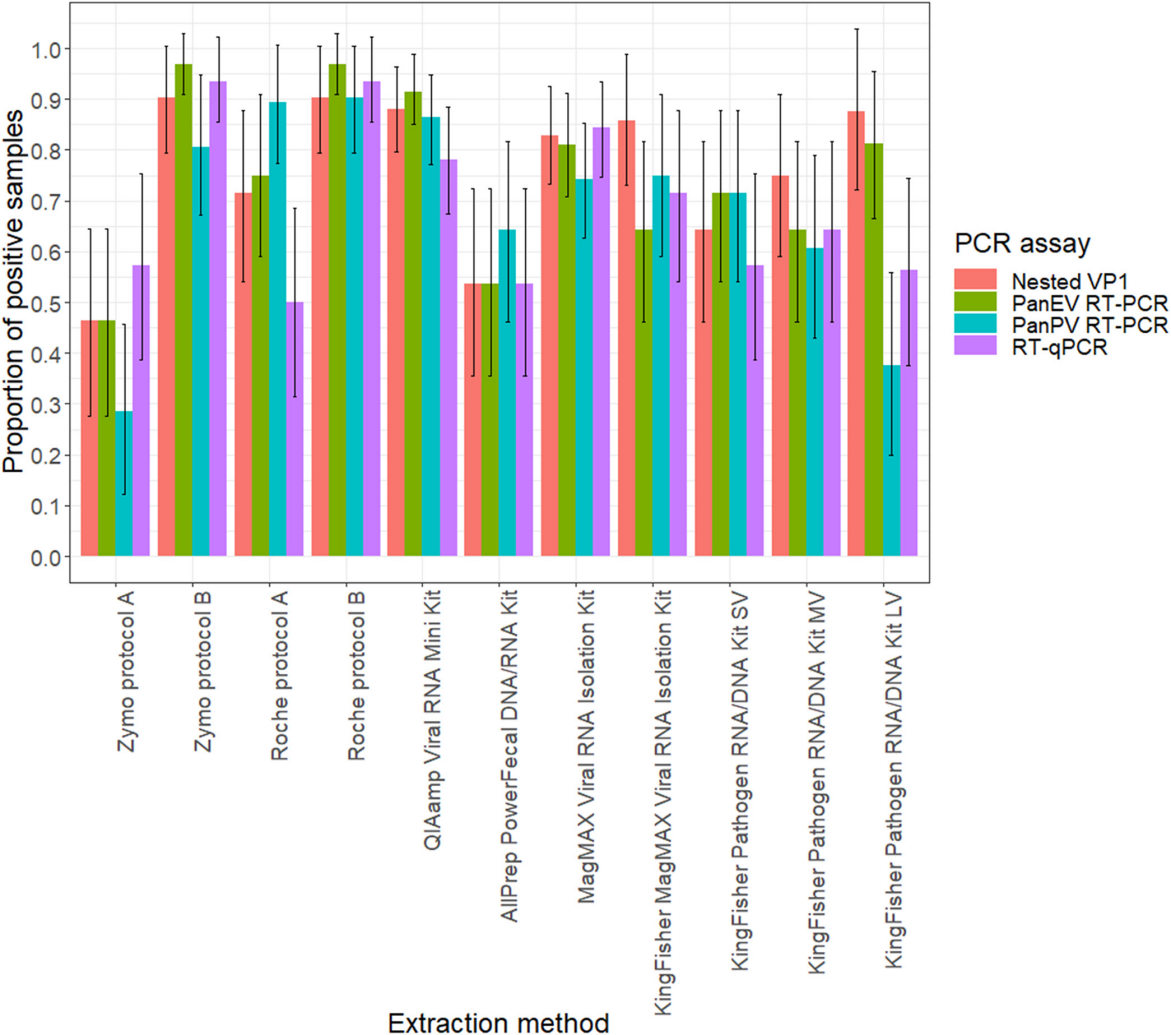
The proportion of PV positive samples recovered by the 11 extraction methods. The black bars indicate the 95% confidence intervals for the true proportion of samples extracted by each extraction method that were PV positive in the different PCR assays (nested VP1, PanEV RT-PCR, PanPV RT-PCR, and RT-qPCR).

The proportion of PV-positive samples in the RT-qPCR, nested VP1, PanEV RT-PCR, and PanPV RT-PCR assays extracted by 11 extraction methods ranged from 0.50 to 0.94, from 0.46 to 0.90, from 0.46 to 0.97, and from 0.29 to 0.90, respectively. The highest proportion of PV positive stool samples in the RT-qPCR was observed in stool samples extracted using the Zymo quick RNA viral kit (protocol B), High Pure viral RNA minikit (protocol B), MagMAX viral RNA isolation kit (manual), and KingFisher MagMAX viral RNA minikit, with results comparable to those obtained with the QIAamp viral RNA minikit (Fig. 2). The lowest proportion of PV positive stool samples in the RT-qPCR was observed in stool samples extracted using the High Pure viral RNA minikit (protocol A), AllPrep PowerFecal DNA/RNA kit, KingFisher MagMAX pathogen RNA/DNA kit (LV), KingFisher MagMAX pathogen RNA/DNA kit (small volume [SV]), and Zymo Quick RNA viral kit (protocol A) (Fig. 2).

The highest proportion of PV positive stool samples for the nested VP1 assay was observed in samples extracted using the Zymo quick RNA viral kit (protocol B), High Pure viral RNA minikit (protocol B), KingFisher MagMAX pathogen RNA/DNA kit (LV), KingFisher MagMAX viral RNA minikit, and MagMAX viral RNA isolation kit (manual), with results comparable to those obtained with the QIAamp viral RNA minikit. The lowest proportion was observed in samples extracted using the Zymo quick RNA viral kit (protocol A), AllPrep PowerFecal DNA/RNA kit, and KingFisher MagMAX pathogen RNA/DNA kit (SV) (Fig. 2). For PV detection using the whole capsid region PanEV RT-PCR assay, the highest proportion of positive samples was observed in those extracted using the Zymo quick RNA viral kit (protocol B), High Pure viral RNA minikit (protocol B), MagMAX viral RNA isolation kit (manual), and KingFisher MagMAX pathogen RNA/DNA kit (LV), with results comparable to those obtained with the QIAamp viral RNA minikit. The lowest proportion was observed in samples extracted using the Zymo quick RNA viral kit (protocol A) and AllPrep PowerFecal DNA/RNA kit (Fig. 2).

The highest proportion of PV-positive stool samples for the whole-genome PanPV RT-PCR assay was observed in samples extracted using the High Pure viral RNA kit (protocol A), High Pure viral RNA minikit (protocol B), Zymo Quick RNA viral kit (protocol B), KingFisher MagMAX viral RNA isolation kit, and MagMAX viral RNA isolation kit (manual), with results comparable to those obtained with the QIAamp viral RNA minikit. The lowest proportion was observed in samples extracted using the Zymo quick viral RNA kit (protocol A) and KingFisher MagMAX pathogen RNA/DNA kit (LV) (Fig. 2).

**FIG 3:**
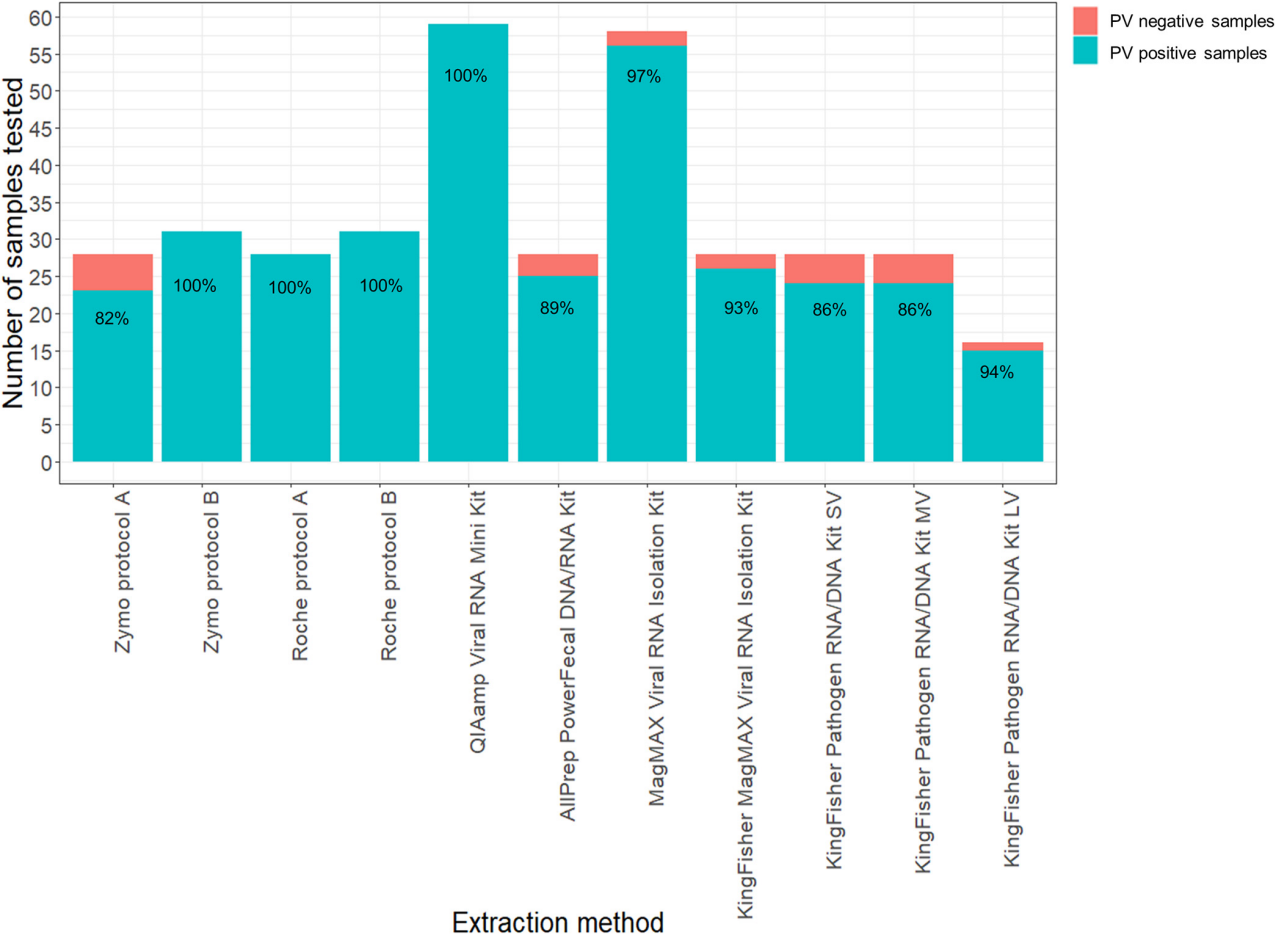
The overall number of samples detected as PV positive and negative for the 11 extraction methods following combined detection with the different PCR assays. The percentage in the bars indicates the sensitivity result for each extraction method after combined PV detection using PV specific RT-qPCR, nested VP1 PCR, and PanEV and PanPV RT-PCR.

All the samples extracted using the High Pure viral RNA kit (protocol A), High Pure viral RNA minikit (protocol B), Zymo quick RNA viral kit (protocol B), and QIAamp viral RNA minikit were successful at yielding PV results in a combination of the different PCR assays (Fig. 3). There was a statistically significant effect of extraction method on the nested VP1, PanEV, and PanPV RT-PCR assays for PV detection (*P* < 0.001). Overall, most methods were not inferior to the QIAamp viral RNA minikit except for the AllPrep PowerFecal DNA/RNA kit, KingFisher MagMAX pathogen RNA/DNA kit (SV), and Zymo quick RNA viral kit (protocol A) (Table S1 in the supplemental material). Additionally, results indicated that some extraction methods proved to be successful at yielding PV results for a given sample, as detected using PV-specific RT-qPCR, nested VP1 PCR, and PanEV and PanPV RT-PCR, while other methods proved to be unsuccessful (Fig. S1 to S4).

### Comparison of the sensitivity of different PCR assays for poliovirus detection.

In the logistic regression analysis that accounted for RNA extraction methods, the different molecular detection assays did not differ significantly in the proportion of samples that were positive (Table S2). In general, performance of the RNA extraction methods was consistent across the different detection assays, although there was a significant negative interaction between the PanPV RT-PCR and the KingFisher MagMAX pathogen RNA/DNA kit (LV), indicating worse performance of this combination of assay and extraction method (*P* < 0.001).

### Effect of increasing the sample volume.

To assess if increasing the input sample volume would increase PV molecular detection particularly for the nested VP1 (a PCR assay that forms part of the DDNS method), three sample volumes were extracted using the MagMAX pathogen DNA/RNA kit (140 μL [small], 300 μL [medium], and 700 μL [large]) on the KingFisher Duo. Comparison of the percentage of all positive samples by the nested VP1 indicated that using a large volume led to a higher percentage of samples that were PV positive than the medium- and small-input sample volume (87.3% versus 78.6% and 64.3%, respectively).

### Processing time and cost comparison.

**FIG 4:**
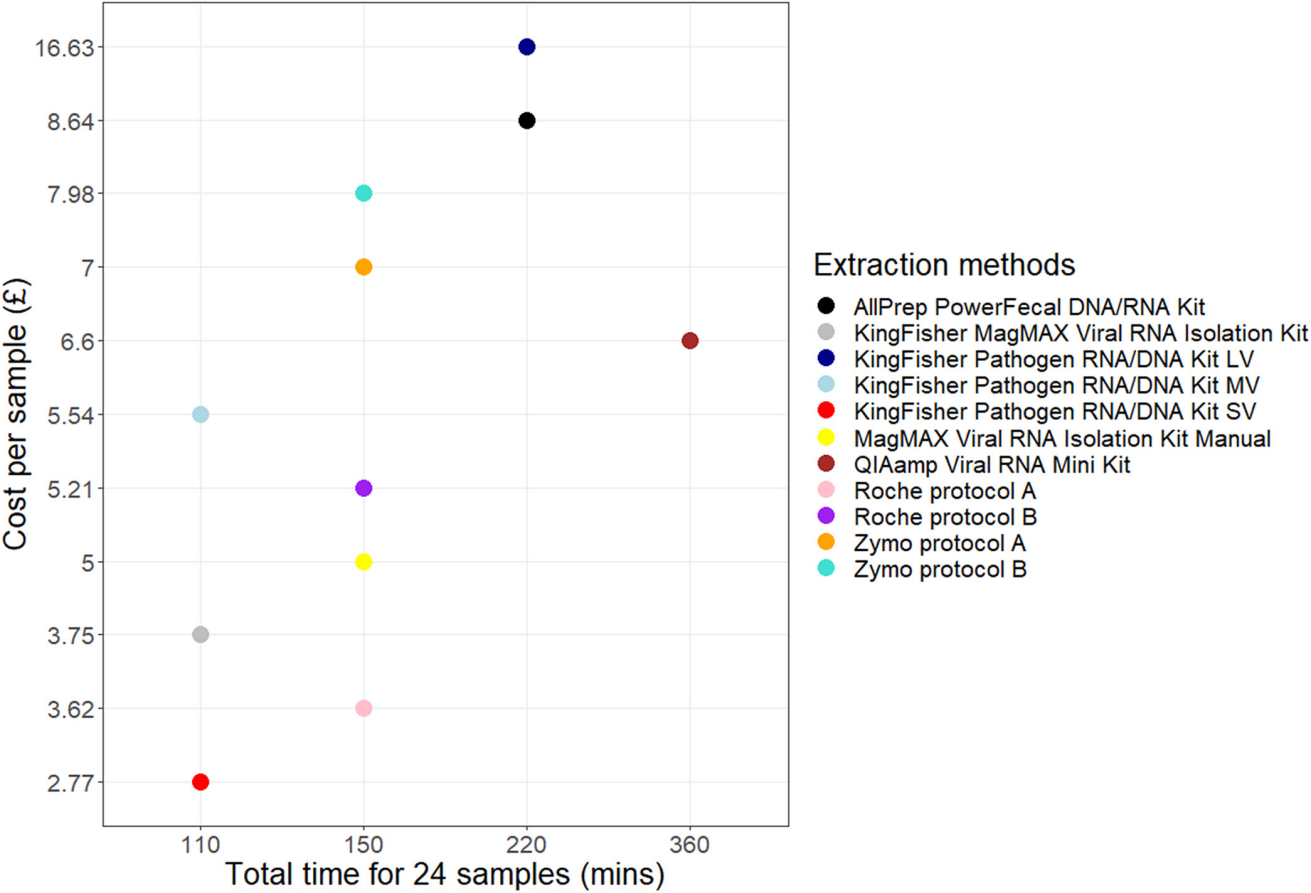
Plot of cost against time for the 11 extraction methods.

The approximate amount of time required to process samples by the 11 extraction methods was determined, and the cost per sample was calculated for the kits and additional reagents required (excluding plasticware) (Fig. 4). The automated extraction methods required the lowest sample processing time. Of the manual extraction methods, the High Pure viral RNA kit (both protocols A and B), MagMAX viral RNA isolation kit, and Zymo quick RNA viral kit (protocols A and B) required the lowest sample processing time. The QIAamp viral minikit, using a 560-μL sample input (four times the manufacturer’s recommended starting input), had the longest processing time of all the 11 extraction methods tested. The lowest cost per sample was obtained for the KingFisher MagMAX pathogen RNA/DNA kit (SV), High Pure viral RNA kit (protocol A), and KingFisher MagMAX viral RNA isolation kit, while the highest cost per sample was obtained for the KingFisher MagMAX pathogen RNA/DNA kit (LV) (Fig. 4).

## DISCUSSION

Efficient direct detection methods for the testing of stool samples from individuals with AFP will be crucial to future poliovirus surveillance. In this study, a comparison of 11 extraction methods (7 manual methods and 4 semiautomated methods) was conducted to assess their ability to recover poliovirus RNA from stool suspensions that had been stored for over 6 months following previous identification as containing poliovirus through virus isolation. All 11 extraction methods assessed use a lysis buffer containing guanidine thiocyanate or guanidine hydrochloride, a component that has been shown to lyse cells and inactivate nucleases (18–20). The RNA recovered by each of the 11 extraction methods was investigated for quality and quantity, and the methods were evaluated for performance using PV-specific RT-qPCR, PanPV RT-PCR that generates a near whole genome, PanEV RT-PCR that amplifies the entire capsid, and nested VP1 PCR that amplifies the full VP1 region.

In terms of RNA quality (RINe) and quantity, the automated extraction methods and MagMAX viral RNA isolation kit (manual) recovered RNA with higher RINe values and higher RNA concentration, respectively, than all the other methods tested in this study. This indicates that magnetic bead-based methods likely recover better-quality RNA than column-based methods, owing to the lack of centrifugation steps through purification columns that can cause nucleic acids to break. Most of the variations in the mean RNA concentration and RINe value observed for the 11 extraction methods can be attributed to the effects of the extraction method used because the matrix effect was controlled for by using the same stool suspension. Although the High Pure viral RNA kit without proteinase K (protocol A) and the Zymo viral RNA kit (protocol B) had the lowest average RINe, indicating low quality RNA, a high proportion of near-whole-genome PV amplicons was observed for RNA extracts recovered with these methods. Moreover, RNA extracts from the Zymo quick RNA minikit (protocol B), High Pure viral RNA minikit with proteinase K (protocol B), QIAamp viral RNA minikit, and MagMAX viral RNA isolation kit that failed to generate a RINe value still showed successful PCR amplification. RINe was found to be statistically significant for only RT-qPCR due to the assay using a much shorter target sequence than other PCR assays performed in this study. This finding suggests that RINe value that reflects total RNA may not necessarily be an indicator of RT-PCR amplification performance.

The results demonstrated that the AllPrep PowerFecal DNA/RNA kit was the worst performer for the molecular detection of enteroviruses, including PV, as the lowest proportion of PV-positive results observed in the RT-qPCR, PanEV RT-PCR, and nested VP1 were found in RNA extracted with this method. No viral contamination was observed in the negative controls used during RNA extraction for all 11 extraction methods. Extraction method was found to have a statistically significant effect on the PanPV RT-PCR, PanEV RT-PCR, and nested VP1 PCR assays. All samples (59/59) in this study extracted by a combination of the different extraction methods and tested by different PCR assays were identified as PV positive by one or more of the PCR assays used. Performing only the PV-specific RT-qPCR and nested VP1 PCR assays would have been sufficient to allow PV detection in all the tested samples. This is not surprising because the RT-qPCR and nested VP1 PCR assays have increased sensitivity due to requiring a very short target to amplify and additional amplification cycles, respectively. Interestingly, we observed that if one extraction method did not yield a PV-positive result for a given sample, often another method proved to be successful. This is vital for PV surveillance, as missed or undetected PV in any given stool sample would increase the risk of undetected circulation, hence delaying the global polio endgame. Overall, results showed that the High Pure viral RNA minikit with proteinase K (protocol B), MagMAX viral RNA isolation kit (both manual and automated methods), Zymo quick RNA minikit (protocol B), and QIAamp viral RNA minikit are better suited for PV molecular direct detection using amplicon-based PCR methods. Although the QIAamp viral RNA minikit performed well, using a 560 μL sample input (four times the manufacturer’s recommended starting input) proved to be labor intensive and time-consuming due to multiple loading of the column. Therefore, it would probably not be ideal for processing medium to large sample sets.

Direct detection by molecular methods for any pathogen is dependent on efficient recovery of high-quality extracted nucleic acid from the sample. Selecting the right extraction method for the research question at hand that also meets the particular laboratory’s needs requires planning and evaluation. Therefore, the following factors should be considered when selecting an extraction method. (i) Sample type used to recover the nucleic acid should be considered. Different sample matrices require different approaches to lysis and extraction, making certain extraction methods more suitable for a given sample type than others (18). (ii) Target nucleic acid type and quality (i.e., RNA or DNA) should also be considered. Recovering high-quality viral RNA suitable for long-range amplification is more challenging than DNA targeted for a short-span PCR, as it is more susceptible to degradation by RNase enzymes, which are abundant in human cells and tissues (12, 18). Many kits copurify RNA and DNA, but specialist kits exist that use special systems and conditions to enable RNA recovery for efficient downstream analysis. (iii) Throughput and cost per sample need to be considered to balance the number of samples to be processed with using financial and human resources efficiently. Nucleic acid extraction methods vary greatly in how long they take to process the same number of samples, how much they cost per sample, and how easily they could be scaled up or down. (iv) Availability, storage conditions, and ease of use are further aspects to contemplate depending on the laboratory setting.

The range of *C_T_* values and the assay cutoff point or the way for determining if a sample is positive or negative should be based on the sensitivity and specificity of the given qPCR assay. These need to be established by testing an appropriate sample panel consisting of negative and positive samples, negative and positive controls, and dilution series of the target. Additionally, a reference method used for comparison should be one that is well established in the literature and widely used for targeting the particular pathogen.

While considering performance, simplicity of the procedure, cost factor, and flexibility to use manually or automatically, we currently consider the MagMAX viral RNA isolation kit to be the preferred method for PV detection in stool samples among the 11 methods compared in this study. Future work could investigate the application of the MagMAX viral RNA isolation kit for PV detection in wastewater, where the sample matrix will differ and where the possibility of processing large-volume sample aliquots could maximize direct detection of the low PV copies present in wastewater. For direct detection of PV by DDNS (using the PanEV RT-PCR followed by the nested VP1 PCRs for nanopore sequencing) to be accepted instead of virus isolation in the polio eradication program, the method would need to be noninferior to virus isolation in terms of sensitivity and specificity and be rapid, robust, easy to use, less expensive, and accessible.

There are limitations to this study. There were insufficient volumes for some of the stool suspensions; hence, not all samples could be extracted by the 11 extraction methods. Relatively old stool samples were used in this study (over 6 months old). Therefore, although all the samples used in this study had been previously identified as PV positive via virus isolation, some were identified as negative by one or more of the PV detection PCR assays used in this study. This suggests that some of the stool samples had lost their integrity. Harrington et al. found PV direct detection following RNA extraction to be significantly more sensitive than virus isolation by cell culture for PV detection in retrospective stool samples (21). In this study, the PV-positive stool suspensions were only tested with direct RNA extraction methods for PV molecular detection. As the number of samples containing a specific PV serotype was relatively small, a conclusion on if PV serotype affected extraction or amplification sensitivity could not be drawn. Moreover, natural stool samples that contained a combination of PV serotypes were used in this study; therefore, the composition between the individual PV serotypes is unknown. Enough samples containing a single PV serotype and/or having them in well-defined combinations would be needed to address whether PV serotype affected extraction or amplification sensitivity, but this was beyond the scope of our study.

In conclusion, this study provides useful information for the selection of RNA extraction methods for stool samples that maximizes their application for PV detection using qPCR or amplicon sequencing, such as the DDNS method (6). The findings indicate that differences in extraction methods for downstream PV molecular direct detection exist and may present various results depending on the PCR amplification assay. The choice of RNA extraction, although often overlooked, is therefore an important component of any direct detection of pathogens in stool.

## MATERIALS AND METHODS

### Samples.

In this study, a sample panel of 59 supernatants from chloroform-treated stool suspensions processed according to the WHO guidelines (22) that had been previously identified as containing polioviruses through virus isolation by cell culture at the National Institute for Health (NIH), Pakistan, were assembled and shipped to the National Institute for Biological Standard Control (NIBSC) laboratory in cold chain and stored at −80°C. Twenty-eight of the Sabin 1 and 3 poliovirus-positive stool suspensions were then transported in cold chain from NIBSC to Imperial College London (ICL) and stored at −80°C. The remaining 31 stool suspensions containing type 2 polioviruses only or in different combinations with Sabin 1 and 3 were processed for testing at NIBSC. Each sample was aliquoted into single microcentrifuge tubes according to the volume required for each RNA extraction method (Table 2) and stored at −80°C until RNA extraction. Some stool suspensions had insufficient volumes to be used in all 11 extraction methods.

**TABLE 2:** Characteristics of the extraction methods

Extraction method^*a*^	Sample input (μL)	Total sample/lysis vol before loading the column/ bead capture (μL)	Sample output (μL)	Concentration factor	Chemistry	Process	Lab
AllPrep PowerFecal DNA/RNA kit	200	3,400	50	4	Spin column	Manual	ICL
High Pure viral RNA kit without proteinase K (Roche protocol A)	200	600	50	4	Spin column	Manual	ICL
High Pure viral RNA kit with proteinase K (Roche protocol B)	200	600	50	4	Spin column	Manual	NIBSC
KingFisher MagMAX viral RNA isolation kit	300	900	50	6	Magnetic beads	Semiautomated	ICL
KingFisher MagMAX pathogen RNA/DNA kit (SV)	140	940	50	2.8	Magnetic beads	Semiautomated	ICL
KingFisher MagMAX pathogen RNA/DNA kit (MV)	300	920	50	6	Magnetic beads	Semiautomated	ICL
KingFisher MagMAX pathogen RNA/DNA kit (LV)	700	4,619	90	7.8	Magnetic beads	Semiautomated	ICL
MagMAX viral RNA isolation kit (manual)	400	1,200	50	8	Magnetic beads	Manual	ICL and NIBSC
QIAamp viral RNA minikit	560	5,040	50	11.2	Spin column	Manual	ICL and NIBSC
Zymo quick RNA viral kit (protocol A)	800	3,200	50	16	Spin column	Manual	ICL
Zymo quick RNA viral kit (protocol B)	400	1,600	25	16	Spin column	Manual	NIBSC

a Methods starting with KingFisher were extracted automatically using the KingFisher Duo instrument. For the KingFisher MagMAX pathogen RNA/DNA kits, SV indicates small volume, MV indicates medium volume, and LV indicates large volume.

### Generation of Sabin 1 standards for genome copy quantification by RT-qPCR.

The RNA from 140 μL of Sabin 1 virus cell culture supernatant was extracted using the QIAamp viral RNA minikit (Qiagen), as per the manufacturer’s instructions. Five microliters of the RNA extract was amplified by a nested RT-PCR, first targeting the entire capsid region using pan-enterovirus primers (8) followed by amplification of the full VP1 region that encompass the qPCR target. The VP1 PCR used 1 μL of unpurified first RT-PCR product with primers Y7 (5′-GGGTTTGTGTCAGCCTGTAATGA-3′) and Q8 (5′-AAGAGGTCTCTRTTCCACAT-3′) (23) and was performed with DreamTaq DNA polymerase (Thermo Scientific) according to the manufacturer’s instructions in a 25-μL reaction volume. The second PCR amplicon product was resolved and visualized on an agarose gel to check for the expected PCR product size (~1,105 bp). The PCR product was then gel excised and purified using the QIAQuick gel extraction kit, as per the manufacturer’s instructions (Qiagen). The length/size and concentration of the purified PCR product was determined using the TapeStation (Agilent Technologies, Inc.), and the copy number of the DNA amplicon template was determined using an online calculator (double-stranded DNA [dsDNA] copy number calculator at http://cels.uri.edu/gsc/cndna.html). A substock standard at 10^7^ copies/μL was made from the purified DNA amplicon and used for the preparation of a 10-fold dilution series in the range 2 × 10^5^ to 2 × 10^1^ copies/μL. The dilutions were aliquoted in 0.2-mL tubes for single use to avoid nucleic acid degradation.

### Nucleic acid extraction.

The extraction methods included seven manual methods (two protocols for the Zymo quick RNA viral kit [Zymo, R1034], two protocols for the High Pure viral RNA kit [Roche, 11858882001], one each for the QIAamp viral RNA minikit [Qiagen, 52904], MagMAX viral RNA isolation kit [Thermo Fisher, AM1939], and AllPrep PowerFecal DNA/RNA kit [Qiagen, 80244]) and two semiautomated extraction methods (MagMAX viral RNA isolation kit [Thermo Fisher, AM1939] and MagMAX Pathogen RNA/DNA kit [Thermo Fisher, 4462359], the latter performed with 140 μL, 300 μL, and 700 μL sample volumes) on the KingFisher Duo instrument (Table 2). The 28 stool suspensions processed at ICL were extracted by 9 of the 11 methods showed in Table 2, while the 31 samples processed at NIBSC were extracted by 4 of the 11 methods shown in Table 2. The selection of the extraction methods was based on their wide commercial use and methods that may favor viral RNA recovery. The Zymo quick RNA viral kit was tested using two methods. At ICL, all the sample was passed through one column with a final elution of 50 μL of nuclease-free water (Zymo protocol A), while at NIBSC, a double extraction procedure in which the sample was split (400 μL each) and extracted in two separate columns was performed, with an elution of 25 μL of nuclease-free water, and the eluates were combined for a final eluate of 50 μL (Zymo protocol B). For the High Pure viral RNA kit extraction method, the protocol used to process samples at NIBSC included enzymatic digestion with proteinase K (Roche protocol B), while proteinase K was not included in the protocol for samples processed at ICL (Roche protocol A). Proteinase K treatment was performed with 50 μL of 20 mg/mL enzyme stock added to the sample and binding buffer mixture at the lysis stage with a 10-min incubation at room temperature. Proteinase K was used for enzymatic inactivation of endogenous nucleases (RNases and DNases) and digestion of contaminating proteins that may be present during nucleic acid extraction, hence aiding recovery and preventing viral RNA degradation (24, 25).

### Determination of RNA quality and concentration.

The RNA quality (RNA Integrity Number equivalent [RINe]) and concentration was determined using RNA ScreenTape and the high-sensitivity RNA ScreenTape assay with 2 μL of extracted RNA performed on the Agilent 2200 or 4200 TapeStation system (Agilent Technologies, Inc.). According to the high-sensitivity RNA TapeStation technical bulletin, RINe values range from 1 to 10, with 1 being the lowest, and a RINe of over 7 expected as an indicator of intact RNA.

### Quantitative one-step RT-PCR (RT-qPCR).

For samples processed at ICL, the extracted RNA for each method was subjected to RT-qPCR with the same primer/probe sets as those provided in the poliovirus intratypic differentiation (ITD) diagnostic real-time reverse transcription PCR (rRT-PCR) version 5.0 kit (US CDC) (26). The reaction was performed using the Promega GoTaq probe one-step RT-qPCR system on the QuantStudio 7 Flex real-time PCR system (Thermo Fisher Scientific, MA, USA). Briefly, 2 μL of extracted RNA was added to 8 μL of master mix containing 5 μL of the GoTaq probe qPCR master mix with dUTP, 0.9 μL of forward and reverse primer each, 0.25 μL of probe, 0.2 μL of GoScript reverse transcriptase mix for one-step RT-qPCR, and 0.75 μL of nuclease-free water. The cycling conditions were 45°C for 30 min (reverse transcription), 95°C for 1 min (reverse transcriptase inactivation), followed by 40 cycles at 95°C for 15 s (denaturation), 42°C for 45 s (annealing), and 60°C for 20 s (extension).

For samples processed at NIBSC, the extracted RNA for each method was subjected to RT-qPCR with poliovirus-specific primers and probe from the Poliovirus rRT-PCR ITD version 5.1 kit using the qScript XLT one-step RT-qPCR Quanta tough mix kit (QuantaBio, Beverly, MA) on the Qiagen Rotor Gene Q platform (5). Briefly, 2 μL of extracted RNA was added to 19 μL of master mix containing 10 μL of tough mix, 1 μL of primer/probe mix, and 8 μL of PCR water. The cycling conditions were 50°C for 30 min (reverse transcription) and 95°C for 1 min (reverse transcriptase inactivation), followed by 40 cycles at 95°C for 15 s (denaturation), 50°C for 45 s (annealing), 61°C for 20 s (extension), and 72°C for 5 s. Unlike the poliovirus probe used in the rRT-PCR screening kit, ITD 5.0 for the pan-poliovirus (PanPV) assay, the PanPV assay probe used in the ITD 5.1 kit has a double quencher (Zen), which reduces background signal and potential false-negative results that are associated with using certain real-time machines, such as the Qiagen Rotor Gene Q (5).

The qPCR was performed in duplicate on material from single RNA extractions. The samples were run in parallel with the Sabin 1 DNA standards to estimate the viral genome copies present in the samples. A *C_T_* value of 40 was assigned to samples that tested negative in the real-time RT-qPCR. To validate comparison of data between qPCR assays, cross-points for the Sabin 1 standards were collected for all qPCR runs and analyzed. There was no significant difference detected between qPCR runs neither at ICL nor at NIBSC, with *R*^2^ ranging from 0.999 to 0.984, a slope of −3.57 to −3.18, and similar deviations, indicating little interrun variability (data not shown).

### Conventional RT-PCR assays.

The extracted viral RNA was subjected to virus detection based on two PCRs. (i) A nested RT-PCR that amplifies the entire capsid region using pan-enterovirus primers (PanEV RT-PCR), followed by full VP1 amplification using degenerate pan-poliovirus primers (nested VP1 PCR used by the DDNS method), as previously described (6, 8). Briefly, 5 μL of RNA extract was amplified using pan-enterovirus primers (8), and 1 μL of the PanEV RT-PCR product was used in the VP1 PCR with barcoded primers (6). (ii) A near-whole-genome poliovirus (PV) amplicon PCR (PanPV RT-PCR) using poliovirus primers PCR F (5′-AGAGGCCCACGTGGCGGCTAG-3′) and PCR 3′ (5′-CCGAATTAAAGAAAATTTACCCCTACA-3′) (27). The PCR products for the PanPV RT-PCR, PanEV RT-PCR, and nested VP1 PCR were resolved and visualized on an agarose gel to confirm the expected PCR product size (~7.5 kb, 3.9 kb, and 1.1 kb, respectively). Samples showing a band at the expected PCR product size were regarded as positive, and those without any band or with a wrong-sized band were identified as negative. The nested RT-PCR forms part of the direct detection by nanopore sequencing (DDNS) method workflow. Therefore, the results obtained from these PCR assays can be used as an indicator of how well the different extraction methods perform with the DDNS method.

### Statistical analyses and plots.

The RT-qPCR and conventional PCR results scored as positive and negative (using a *C_T_* threshold of 40 for the RT-qPCR) were analyzed using a logistic regression model (estimated using maximum likelihood).The model included sample ID as a random effect on the intercept, and QIAamp viral RNA minikit was used as the reference group, as it showed good overall performance in all assays, and it included all samples that were extracted by the methods tested in this study. To compare the sensitivity of the 11 extraction methods, the proportion of PV-positive samples as identified by each PV direct detection PCR assay was determined along with the 95% confidence intervals. We also compared log viral copy numbers estimated using RT-qPCR using a linear mixed effects regression with sample ID as a random effect. *P* values of ≤0.05 were considered statistically significant. All analyses were performed in R environment, version 4.1.3.
