# Supplementary material for: Comparison of Eleven RNA Extraction Methods for Poliovirus Direct Molecular Detection in Stool Samples
Source: Microbiol Spectr. 2023 Mar 20;11(2):e04252-22. doi: 10.1128/spectrum.04252-22 (PMC10100708; doi:10.1128/spectrum.04252-22)
Supplement: Supplemental file 1 — supplemental material. Download spectrum.04252-22-s0001.pdf, PDF file, 0.3 MB [file spectrum.04252-22-s0001.pdf]

Supplementary Material

Comparison of eleven RNA extraction methods for poliovirus direct molecular detection in stool samples

Joyce Odeke Akello[a#], Erika Bujaki[b], Alexander G. Shaw[a], Adnan Khurshid[c], Yasir Arshad[c], Catherine Troman[a], Manasi Majumdar[b], Áine O'Toole[d], Andrew Rambaut[d], Muhammad Masroor Alam[c], Javier Martin[b], Nicholas C. Grassly[a]

[a]Department of Infectious Disease Epidemiology, Imperial College London, London , United kingdom

[b]Division of Vaccines, National Institute for Biological Standards and Control (NIBSC), MHRA, Potters Bar, United Kingdom

[c]Department of Virology, National Institute for Health, Islamabad, Pakistan

[d]Institute of Evolutionary Biology, University of Edinburgh, Ashworth Laboratories, Edinburgh, United Kingdom

Running Head: RNA extraction methods for poliovirus direct detection

#Address correspondence to Joyce Odeke Akello, (j.akello@imperial.ac.uk).

Joyce Odeke Akello and Erika Bujaki contributed equally to this work.

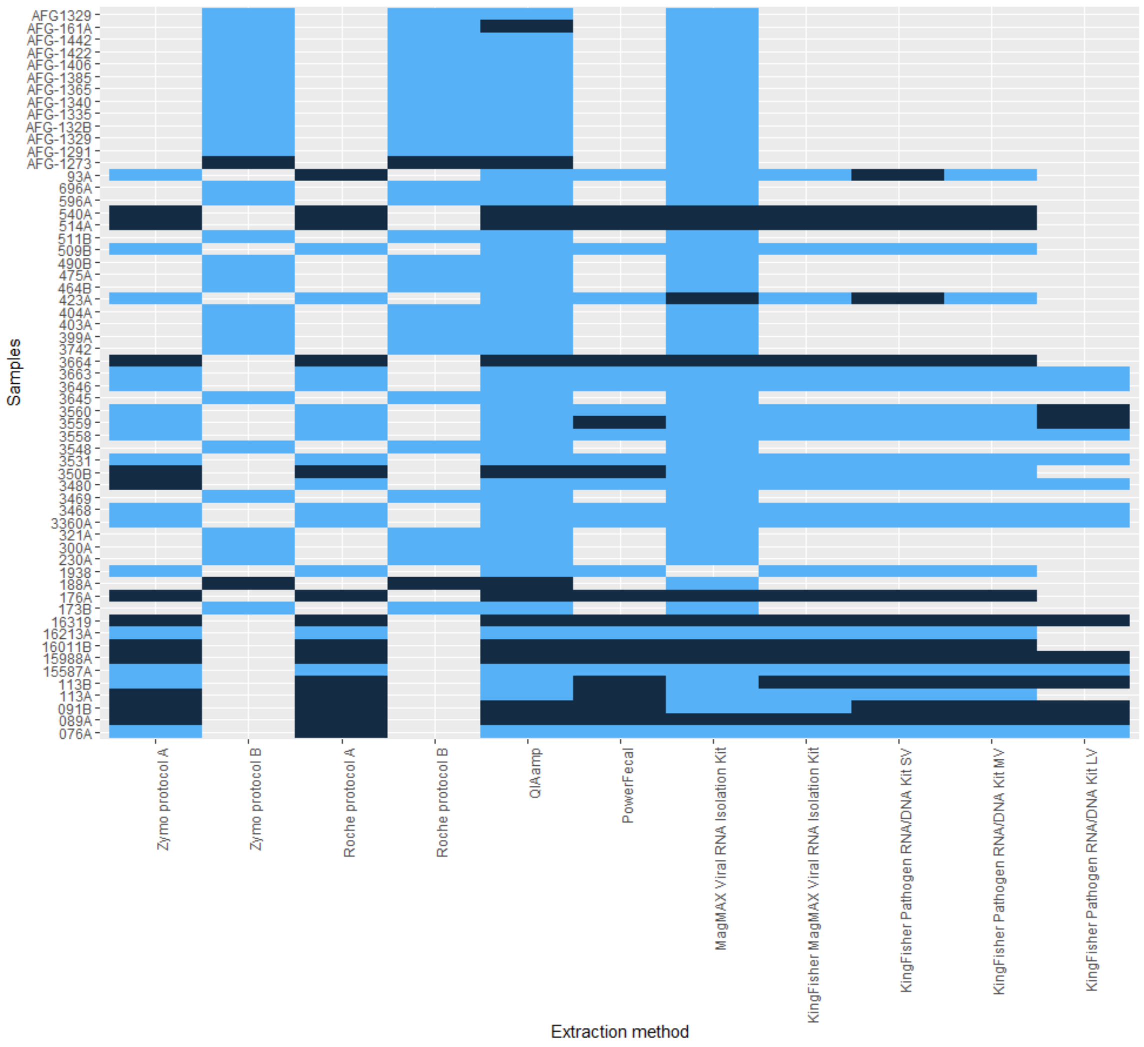

Figure S1. Heatmap showing samples that were identified as poliovirus positive or negative in the poliovirus specific RT-qPCR by each of the eleven extraction methods tested in this study. Positive result represented by blue, negative by black, and the grey indicates that a particular sample was not extracted by that method. The results indicated that some samples were missed / undetected for poliovirus when extracted with certain extraction methods. The extraction methods conducted at NIBSC included the Zymo protocol B, Roche protocol B, QIAamp and MagMAX Viral RNA Isolation kit. The extraction methods conducted at ICL included the Zymo protocol A, Roche protocol B,

QIAamp, PowerFecal, MagMAX Viral RNA Isolation Kit, KingFisher MagMAX Viral RNA Isolation Kit, and the KingFisher Pathogen RNA/DNA Kits (SV, MV and LV)

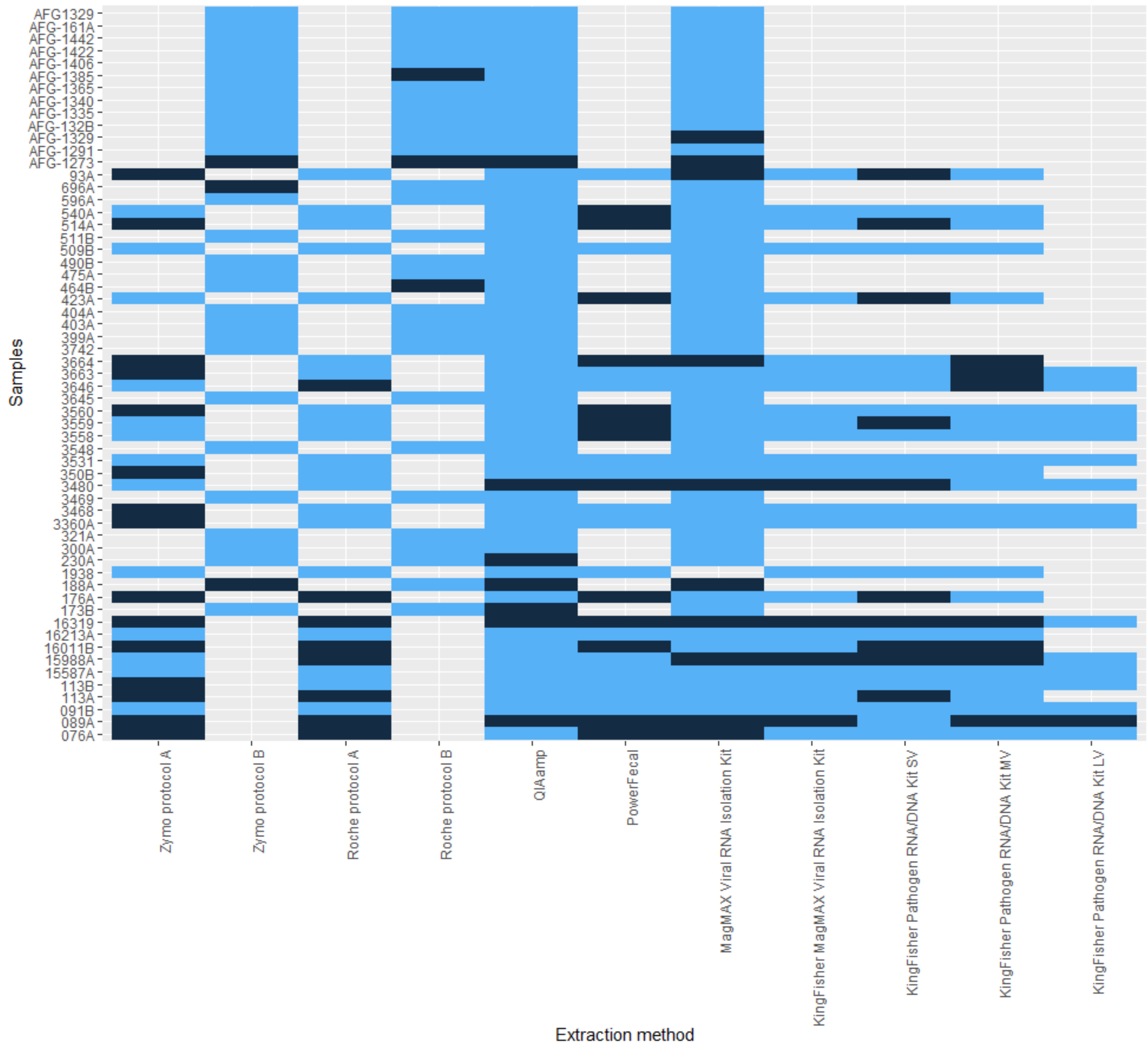

Figure S2. Heatmap showing samples that were identified as poliovirus positive or negative in the nested VP1 PCR by each of the eleven extraction methods tested in this study. Positive result represented by blue, negative by black, and the grey indicates that a particular sample was not extracted by that method. The results indicated that some samples were missed / undetected for poliovirus when extracted with certain extraction methods. The extraction methods conducted at NIBSC included the Zymo protocol B,

Roche protocol B, QIAamp and MagMAX Viral RNA Isolation kit. The extraction methods conducted at ICL included the Zymo protocol A, Roche protocol B, QIAamp, PowerFecal, MagMAX Viral RNA Isolation Kit, KingFisher MagMAX Viral RNA Isolation Kit, and the KingFisher Pathogen RNA/DNA Kits (SV, MV and LV)

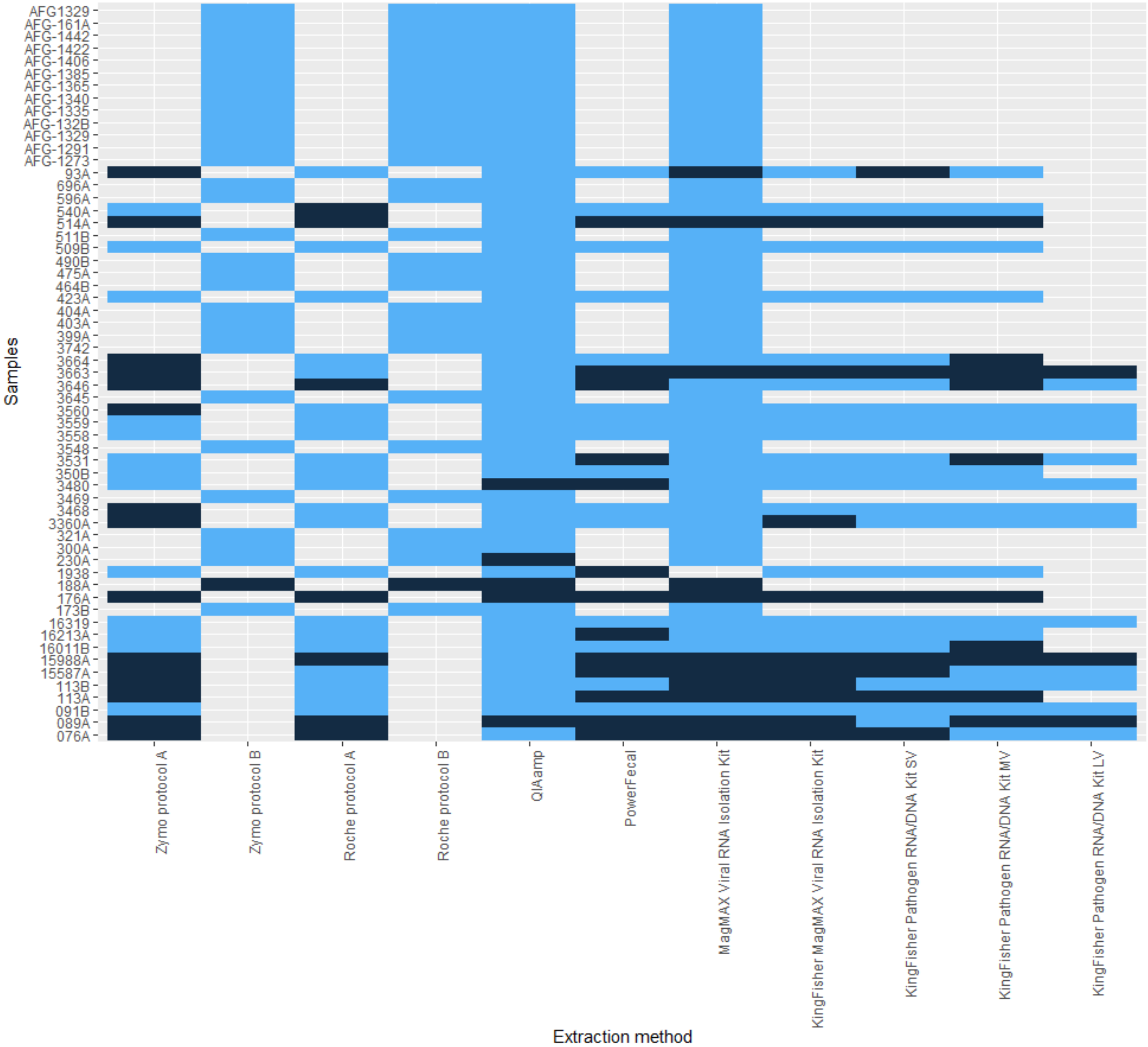

Figure S3. Heatmap showing samples that were identified as poliovirus positive or negative in the PanEV RT-PCR by each of the eleven extraction methods tested in this study. Positive result represented by blue, negative by black, and the grey indicates that a particular sample was not extracted by that method. The extraction methods conducted at

NIBSC included the Zymo protocol B, Roche protocol B, QIAamp and MagMAX Viral RNA Isolation kit. The extraction methods conducted at ICL included the Zymo protocol A, Roche protocol B, QIAamp, PowerFecal, MagMAX Viral RNA Isolation Kit, KingFisher MagMAX Viral RNA Isolation Kit, and the KingFisher Pathogen RNA/DNA Kits (SV, MV and LV)

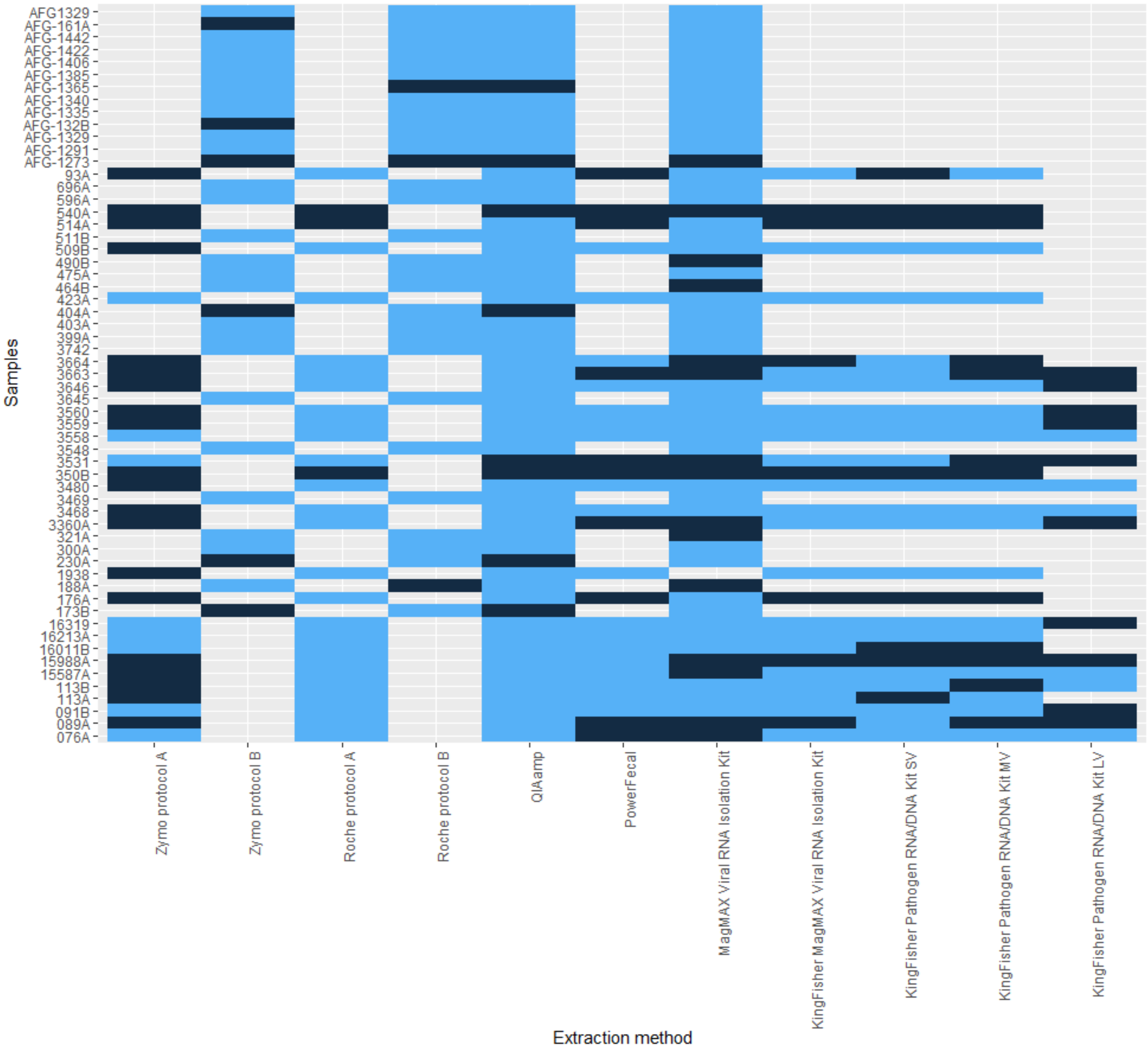

Figure S4. Heatmap showing samples that were identified as poliovirus positive or negative in the PanPV RT-PCR by each of the eleven extraction methods tested in this study. Positive result represented by blue, negative by black, and the grey indicates that a

particular sample was not extracted by that method. The extraction methods conducted at NIBSC included the Zymo protocol B, Roche protocol B, QIAamp and MagMAX Viral RNA Isolation kit. The extraction methods conducted at ICL included the Zymo protocol A, Roche protocol B, QIAamp, PowerFecal, MagMAX Viral RNA Isolation Kit, KingFisher MagMAX Viral RNA Isolation Kit, and the KingFisher Pathogen RNA/DNA Kits (SV, MV and LV)

Table S1: Logistic regression model results for the effect of extraction methods on PCR assays for PV detection.

	Nested VP1				PanEV RT-PCR				PanPV RT-PCR			
**Random effects**	**σ2**	**SD**			**σ2**	**SD**			**σ2**	**SD**		
Sample (Intercept)	0.042	0.204			0.065	0.254			0.053	0.231		
Residual	0.109	0.330			0.085	0.291			0.120	0.346		
**Fixed effects**	**b**	**SE**	**t - value**	**p - value**	**b**	**SE**	**t - value**	**p - value**	**b**	**SE**	**t - value**	**p - value**
(Intercept)	0.881	0.050	17.457	**<.001**	0.915	0.050	18.195	**<.001**	0.864	0.054	15.970	**<.001**
AllPrep PowerFecal RNA/DNA Kit	-0.331	0.079	-4.213	**<.001**	-0.321	0.070	-4.563	**<.001**	-0.203	0.083	-2.462	**0.014**
High Pure Viral RNA kit without proteinase K (protocol A)	-0.152	0.079	-1.939	**0.053**	-0.107	0.070	-1.515	0.136	0.047	0.083	0.564	0.573
High Pure Viral RNA kit with proteinase K (protocol B)	0.008	0.076	0.112	0.911	-0.001	0.068	-0.008	0.993	0.022	0.079	0.282	0.778
KingFisher MagMAX Viral RNA Isolation kit	-0.009	0.079	-0.120	0.905	-0.214	0.070	-3.039	**0.003**	-0.096	0.083	-1.165	0.245
KingFisher Pathogen RNA/DNA Kit SV	-0.224	0.079	-2.849	**0.005**	-0.142	0.073	-2.023	**0.044**	-0.132	0.083	-1.597	0.111
KingFisher Pathogen RNA/DNA Kit MV	-0.117	0.079	-1.484	0.139	-0.214	0.070	-3.039	**0.003**	-0.239	0.083	-2.894	**0.004**
KingFisher Pathogen RNA/DNA Kit LV	0.087	0.097	0.901	0.368	-0.027	0.086	-0.313	0.754	-0.527	0.102	-5.188	**<.001**
MagMAX Viral RNA Isolation Kit	-0.050	0.061	-0.821	0.412	-0.103	0.054	-1.911	0.057	-0.121	0.064	-1.890	0.060
Zymo Quick RNA Viral kit (protocol A)	-0.402	0.079	-5.123	**<.001**	-0.392	0.070	-5.579	**<.001**	-0.561	0.083	-6.785	**<.001**
Zymo Quick RNA Viral kit (protocol B)	0.008	0.076	0.112	0.911	-0.001	0.068	-0.008	0.993	-0.074	0.079	-0.936	0.350

Table S2: Multivariable logistic mixed effects model including RNA extraction method, PCR assay and their interaction.

Random effects	σ2	SD			
Sample (Intercept)	0.045	0.212			
Residual	0.122	0.350			
**Fixed effects**	**Estimate**	**Std. error**	**df**	**t - value**	**p - value**
(Intercept)	0.881	0.053	516.69	16.567	< 2e-16
AssayPanEV	0.034	-0.064	1350.27	0.527	0.599
AssayPanPV	-0.017	-0.064	1350.27	-0.263	0.792
AssayqPCR	-0.102	-0.064	1350.27	-1.580	0.114
ExtractionKingFisher MagMAX Viral RNA Isolation kit	0.036	0.082	1372.99	0.437	0.662
ExtractionKingFisher Pathogen RNA/DNA Kit LV	0.086	0.100	1367.70	0.858	0.391
ExtractionKingFisher Pathogen RNA/DNA Kit MV	-0.072	0.082	1372.99	-0.877	0.381
ExtractionKingFisher Pathogen RNA/DNA Kit SV	-0.179	0.082	1372.99	-2.191	0.029
ExtractionMagMAX Viral RNA Isolation Kit	-0.050	0.065	1350.36	-0.780	-0.436
ExtractionAllPrep PowerFecal RNA/DNA Kit	-0.286	0.082	1372.99	-3.505	< .001
ExtractionHigh Pure Viral RNA kit without proteinase K (protocol A)	-0.107	0.082	1372.99	-1.315	0.189
ExtractionHigh Pure Viral RNA kit with proteinase K (protocol B)	-0.032	0.079	1370.47	-0.409	0.683
ExtractionZymo Quick RNA Viral kit (protocol A)	-0.357	0.082	1372.99	-4.380	< .001
ExtractionZymo Quick RNA Viral kit (protocol B)	-0.032	0.079	1370.468	-0.409	0.683
AssayPanEV: ExtractionKingFisher MagMAX Viral RNA Isolation kit	-0.248	0.113	1350.27	-2.188	0.029
AssayPanPV:ExtractionKingFisher MagMAX Viral RNA Isolation kit	-0.090	0.113	1350.27	-0.795	0.427
AssayqPCR: ExtractionKingFisher MagMAX Viral RNA Isolation kit	-0.077	0.113	1350.27	-0.678	0.498
AssayPanEV: ExtractionKingFisher Pathogen RNA/DNA Kit LV	-0.159	0.139	1350.27	-1.140	0.254
AssayPanPV:ExtractionKingFisher Pathogen RNA/DNA Kit LV	-0.546	0.139	1350.27	-3.915	< .001
AssayqPCR: ExtractionKingFisher Pathogen RNA/DNA Kit LV	-0.273	0.139	1350.27	-1.961	0.050
AssayPanEV: ExtractionKingFisher Pathogen RNA/DNA Kit MV	-0.141	0.113	1350.27	-1.243	0.211
AssayPanPV: ExtractionKingFisher Pathogen RNA/DNA Kit MV	-0.126	0.113	1350.27	-1.110	0.267
AssayqPCR: ExtractionKingFisher Pathogen RNA/DNA Kit MV	-0.005	0.113	1350.27	-0.048	0.962
AssayPanEV: ExtractionKingFisher Pathogen RNA/DNA Kit SV	0.038	0.113	1350.27	0.331	0.741
AssayPanPV:ExtractionKingFisher Pathogen RNA/DNA Kit SV	0.088	0.113	1350.27	0.779	0.436
AssayqPCR: ExtractionKingFisher Pathogen RNA/DNA Kit SV	0.030	0.113	1350.27	0.267	0.790
AssayPanEV: ExtractionMagMAX Viral RNA Isolation Kit	-0.051	0.091	1350.27	-0.559	0.576
AssayPanPV: ExtractionMagMAX Viral RNA Isolation Kit	-0.069	0.091	1350.27	-0.758	0.449
AssayqPCR: ExtractionMagMAX Viral RNA Isolation Kit	0.119	0.091	1350.27	1.301	0.193
AssayPanEV: ExtractionAllPrep PowerFecal RNA/DNA Kit	-0.034	0.113	1350.27	-0.299	0.765
AssayPanPV: ExtractionAllPrep PowerFecal RNA/DNA Kit	0.124	0.113	1350.27	1.094	0.274
AssayqPCR: ExtractionAllPrep PowerFecal RNA/DNA Kit	0.1	0.113	1350.27	0.896	0.370
AssayPanEV: ExtractionHigh Pure Viral RNA kit without proteinase K (protocol A)	0.002	0.113	1350.27	0.016	0.987
AssayPanPV: ExtractionHigh Pure Viral RNA kit without proteinase K (protocol A)	0.196	0.113	1350.27	1.723	0.085
AssayqPCR: ExtractionHigh Pure Viral RNA kit without proteinase K (protocol A)	-0.113	0.113	1350.27	-0.992	0.321

Continuation Table S2: Multivariable logistic mixed effects model including RNA extraction method, PCR assay and their interaction.

Fixed effects	Estimate	Std. error	df	t - value	p - value
AssayPanEV: ExtractionHigh Pure Viral RNA kit with proteinase K (protocol B)	0.031	0.110	1350.27	0.279	0.780
AssayPanPV: ExtractionHigh Pure Viral RNA kit with proteinase K (protocol B)	0.017	0.110	1350.27	0.155	0.877
AssayqPCR: ExtractionHigh Pure Viral RNA kit with proteinase K (protocol B)	0.134	0.110	1350.27	1.221	0.222
AssayPanEV: ExtractionZymo Quick RNA Viral kit (protocol A)	-0.034	0.113	1350.27	-0.299	0.765
AssayPanPV: ExtractionZymo Quick RNA Viral kit (protocol A)	-0.162	0.113	1350.27	-1.425	0.155
AssayqPCR: ExtractionZymo Quick RNA Viral kit (protocol A)	0.209	0.113	1350.27	1.841	0.066
AssayPanEV: ExtractionZymo Quick RNA Viral kit (protocol B)	0.031	0.110	1350.27	0.279	0.780
AssayPanPV: ExtractionZymo Quick RNA Viral kit (protocol B)	-0.080	0.110	1350.27	-0.728	0.467
AssayqPCR: ExtractionZymo Quick RNA Viral kit (protocol B)	0.134	0.110	1350.27	1.221	0.222
